# Spontaneous quantitative processing in Chinese singular and plural picture naming: An event-related potentials analysis

**DOI:** 10.3389/fnins.2022.898526

**Published:** 2022-10-11

**Authors:** Li-yan Cui, Wen-wen Cheng, Sha-rui Shan, Wen Lv, Chen-ming Sun, Run Li, Shu Zhou, Zhuo-ming Chen, Sheng-yong Bao

**Affiliations:** ^1^Department of Rehabilitation Medicine, Shenzhen People’s Hospital, The Second Clinical Medical College, Jinan University, The First Affiliated Hospital, Southern University of Science and Technology, Shenzhen, Guangdong, China; ^2^Department of Rehabilitation Medicine, The First Affiliated Hospital of Jinan University, Guangzhou, China; ^3^Department of Neurology, Maoming People’s Hospital, Maoming, China; ^4^Department of Rehabilitation Medicine, The First Affiliated Hospital of Guangdong Pharmaceutical University, Guangzhou, China; ^5^Department of Neurology, Nanfang Hospital, Southern Medical University, Guangzhou, China

**Keywords:** picture naming, singular, plural, word production, quantitative processing, event-related potentials (ERP), Chinese

## Abstract

Chinese nouns lack inflection and cannot reflect the quantitative relationship between singular and plural numbers. However, neural processes of picture naming are different from those of words. We assume that Chinese single and plural picture naming is different, and they may involve quantitative processing. Therefore, Experiment 1 was designed by picking picture naming as the task and Chinese as the target language and compared the accuracy, reaction time, and event-related potentials (ERPs) between single and plural picture naming, where two types of pictures were mixed. Although the *T*-test showed no significant differences in behavioral data, there were differences in ERPs. ERP differences involved two effects: P1 of 160–180 ms and P2 of 220–260 ms in the parietal-occipital lobe. These differences are suggested to reflect the neural differences in quantitative processing. Therefore, Chinese singular and plural picture naming consists of word production and implicit quantitative processing simultaneously. To explore the relationship between the two processings, we added a semantic factor (inanimate vs. animate items) to the quantity factor of Experiment 1 and carried out Experiment 2, with the observation indexes unchanged. There were no significant differences in behavioral data among the four conditions. After variance analysis, ERPs results indicated an interaction between semantic and quantitative factors in the central area at 180–280 ms. In summary, we suggest that Chinese singular and plural picture naming includes two simultaneous neural processing tasks: word production and quantitative processing, which interact in the central area at 180–280 ms.

## Introduction

Pictures become symbols of their objects by physical similarity. Several studies propose that recognizing images involves consistent cognitive processes as are applied when perceiving real objects (Potter, 1979; Glaser, 1992). And picture naming comprises the same process of conceptually driven word production (Glaser, 1992; Bock and Levelt, 1994). Numerous studies have investigated the neuropsychological process of picture naming and word production using various methods, including functional magnetic resonance imaging (fMRI) (van Turennout et al., 2000), magnetoencephalography (MEG) (Salmelin et al., 1994), positron emission tomography (PET) (Papathanassiou et al., 2000), electroencephalogram (EEG) (Hassan et al., 2015), etc. It is evidenced that picture naming and word production share the same temporal and spatial signatures (Indefrey and Levelt, 2004). Most studies support the “Lemma model” of lexical access proposed by Levelt et al. (1999), which divides word production into six steps: visual extraction, lexical concept formation, lemma selection, phonological coding, phonological words coding, and pronunciation. Therefore, picture naming is an important experimental paradigm in cognitive psychology.

A recent study, which tracked spatiotemporal dynamics networks of picture naming cognitive activity, applied EEG source connectivity analysis to further elucidate the “Lemma model” (Hassan et al., 2015). From the sight of pictures to the completion of articulation, picture naming comprises six brain network states (BNSs). BNS1 (0–119 ms), mainly involving the inferior occipital, is related to visual feature extraction. BNS2 (120–150 ms), primely comprising occipital regions, is responsible for visual information process and object recognition. BNS3 (151–190 ms) indicates lexical retrieval, lemma retrieval, and lemma selection occurrence at the occipital and bilateral inferior temporal sulcus. BNS4 (191–320 ms) spreads to the left inferior temporal gyrus for integrating access to phonological forms. BNS5 (321–480 ms), mainly involving precentral, is responsible for phonetics and articulation. In addition to articulation, BNS6 (481–535 ms) may be related to introspection over the left insular gyrus.

Based on most studies on the neuropsychological process of picture naming were carried on a single object/image, it is generally believed that picture naming is equivalent to the word production process of the “Lemma model.” However, in daily life, objects are more often presented in a plural form than in a singular form (Schiller and Caramazza, 2003). At present, from the perspective of neuro mechanism, there is no research focusing on the naming of singular and plural pictures. Only a few articles have studied the differences between singular and plural pictures in behavioral experiment, but the argument lies in the activation of lexico-syntactic (Khwaileh et al., 2015; Beyersmann et al., 2018). Several articles have studied the quantitative concept of singular and plural pictures with a congruent or incongruent quantifier, founding that the number feature of pictures is different from the word production process and they were not a competitive relationship (Schiller and Caramazza, 2002; Arcara et al., 2019). Most studies focused on the word production process of singular and plural nouns. The research materials are often phrases or sentences, and the focus is mainly on inflectional grammar (Sahin et al., 2009; Gimenes and Brysbaert, 2016). These studies have shown that the word production processes of singular and plural nouns are different due to inflection. Given these, we wondered whether the procedure of singular picture naming would be different from that of plural picture naming.

Numerical magnitude is an abstract quality of a set. It can be represented in a symbolic or non-symbolic form (“10,” “ten,” and “••••••••••”) (Holloway and Ansari, 2010). The singular (the set “1”) opposed to the plural, which encompasses all other numbers treated as a whole (the set “2, 3, 4,…”), has a different quantitative meaning (Hodent et al., 2005). Accumulating studies suggest that the ability of non-verbal representation of numerical magnitudes is native (Starkey and Cooper, 1980; Wynn, 1992; Butterworth, 2005), as it is shown that very young infants and newborns can distinguish syllables, moving objects, collections of objects, and simple dots (Antell and Keating, 1983; Wynn, 1992; Wynn et al., 2002; McCrink and Wynn, 2004). A similar phenomenon is also found in animals (Wynn, 1992; Romo et al., 1999). Further research has indicated that this not only involves a perceptual pattern but also arithmetical operations (Wynn et al., 2002; McCrink and Wynn, 2004). Even if continuous variables (such as area and contour length of items) are controlled, in a looking-time procedure on numbers of items, a 5-month baby did not simply expect “more” or “less” than the initial number of items seen but rather expected exactly the correct number of items (for example, perform 1 + 1 or 2 – 1 by Mickey toys, the baby looking more time on the wrong numbers of toys). This supports that the nature of human infants’ numerical knowledge is based on the accumulator mechanism (magnitude-based estimation system) proposed by Meck and Church (1983); Gallistel and Gelman (1992), Wynn et al. (2002), and McCrink and Wynn (2004) but not an automatic object-tracking mechanism (Kahneman et al., 1992; Trick and Pylyshyn, 1994).

In 1993, Dehaene and Changeux proposed a classic neural processing model of quantitative processing based on non-symbolic numbers (Dehaene and Changeux, 1993). First, objects of different sizes and positions are input and characterized by the retina. Then, the sizes and positions are standardized through a topological map formed by a fixed group of neurons. Finally, the quantitative detector summarizes all outputs and forms a neural map that is highly correlated with quantity. Verguts and Fias further developed this model and applied it to the processing of symbol numbers (Verguts and Fias, 2004). Both computational models focus on sequentially occurring summation coding and spatial coding, which characterize quantitative information of objects to the quantitative processing neural network. While the summation coding assumes that neuronal activity increases linearly with an increasing number (Nieder and Merten, 2007; Roitman et al., 2007; Santens et al., 2010), the spatial coding assumes that certain neurons are associated with specific numbers, suggesting that neuron activation is logarithmic to number, and they generate maximum activation for the number of preferences (Nieder et al., 2002, 2006; Piazza et al., 2004). It is believed that the selection of the coding methods is task-dependent (Parker and Newsome, 1998; Salinas, 2006). The summation coding may be preferred in the number comparison task (Romo et al., 1999) and the spatial coding may be preferred in the discrepancy comparison task (Nieder and Merten, 2007). Based on these two computational models, computer simulations can account for several phenomena in the numerical domain, including the distance effect and Fechner’s law for numbers (Verguts and Fias, 2004). The computer simulations may also demonstrate that human infants and several animal species possess some elementary abilities for numerical processing or calculation, despite the lack of language or task acknowledgment (Verguts and Fias, 2004). Therefore, basic numerical abilities are natural and native. Quantity processing may be widely present in daily life in a subliminal manner when one subject is not aware of having seen objects or a number symbol (Dehaene et al., 1998; Naccache and Dehaene, 2001). Some studies have also found quantitative processing under non-computing tasks (Roitman et al., 2007).

If picture naming is the same as the process of word production. In a language with inflection (such as English), the neural processing of singular and plural picture naming are markedly different because the morphologies and pronunciations of naming have altered, according to the “lemma model” (e.g., basketball vs. basketballs/mouse vs. mice) (Sahin et al., 2009). Interestingly, we found that the word productions of singular and plural pictures in a language without inflection (such as Chinese) were consistent (e.g., Lan Qiu vs. Lan Qiu) (Yu et al., 2013). In other words, Chinese singular and plural picture namings share the same neural processings. However, we know that the essence of inflection in nouns is to indicate the number of objects. And when pictures were presented, we could visually clearly see the number difference of singular and plural objects, even if Chinese lack inflection. Boldly, we doubted that we could even perceive the difference in quantity. Based on these, we proposed a hypothesis: singular and plural picture naming may include both word production and quantitative processing. In other words, singular and plural pictures are still quantitatively processed under the task of naming. According to this theory, the neural processing of singular and plural picture namings are different, at least in quantitative processing. It will bring about a great challenge to the traditional treatment method of picture naming for Chinese aphasia, and even the picture learning method for Chinese children (using singular pictures). And the concept of quantitative processing may also provide a new theoretical direction for further research on the mechanism of picture naming.

The inflection of English would interfere with our judgment on the existence and characteristics of quantitative processing. However, in case neural processes of Chinese singular and plural picture namings are different, it will probably be due to the quantitative processing. Therefore, to prove our hypothesis, Experiment 1 was designed, which used event-related potential (ERP) technology and Chinese picture naming task to compare neural processes of singular and plural pictures. At the same time, we assume that the two processes in one task could not be unrelated, so Experiment 2 was conducted to explore the relationship between them.

## Experiment 1

### Study design

#### Participants

Twenty young healthy postgraduate students (8 male; aged 22–30 years old, and mean age = 25.2 years old, SD: 2.48 years) from Jinan University took part in this experiment as paid participants. These subjects were right-handed, native Chinese speakers. All reported having no previous history of neurological, reading, or learning disorders. All had normal or corrected-to-normal vision. All participants signed written informed consent after all the experimental procedures were fully explained. The study was approved by the Medical Ethics Committee of the First Affiliated Hospital of Jinan University.

#### Stimuli

**FIGURE 1:**
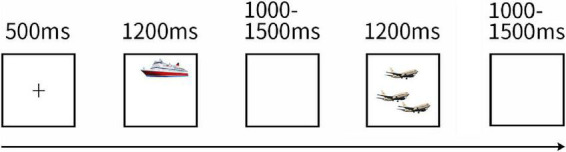
Task Design of Experiment 1. The picture shows the design of the experimental task. All trials followed the depicted sequence. A block began with a fixation cross a picture. Then a singular/plural picture was displayed and participants were asked to name the objects rapidly after the stimuli were presented, followed by a blank screen (three pictures with similar pronunciation or the same type (singular/plural) did not appear consecutively).

The linguistic stimuli were 66 concrete, countable, inanimate, and different objects, which were adopted from the Ni’picture database (Ni et al., 2019; Supplementary Appendix 1). They consisted of two types of pictures: 33 pictures representing one object (singular pictures) and 33 pictures representing three objects (plural pictures). The properties of the two types of items were matched in object familiarity, visual complexity, name agreement, image agreement, image variability, age of acquisition, and word frequency (see Table 1). The 66 pictures from Ni’picture database were edited by Adobe Photoshop CC 2018. They were all set 1,000 × 1,000 pixels, not changing the color and distinguishability of objects. The size of an object in a singular picture was 30,000 pixels, and each size of the object in a plural picture was 10,000 pixels. Objects were placed randomly in both types of pictures (see Figure 1).

**TABLE 1:** Mean (SD) scores for the list of 132 pictures; 33 singular-inanimate (Q−S−), 33 plural–inanimate (Q + S−), 33 singular-animate (Q−S +), and 33 plural-animate (Q + S +).

Properties Type	Q−S−	Q + S−	Q−S +	Q + S +	*F*	*P*
**N-A**	0.78(0.18)	0.76(0.18)	0.68(0.20)	0.75(0.18)	1.826	0.146
**Im-A**	3.97(0.51)	4.03(0.67)	3.80(0.53)	3.89 (0.53)	0.991	0.399
**Fam**	3.11(0.65)	3.09(0.80)	3.09(0.93)	3.15(0.81)	0.044	0.988
**Vi-C**	2.44(0.94)	2.47(0.93)	2.70(0.68)	2.64(0.61)	0.828	0.481
**Im-V**	2.13(0.65)	2.20(0.74)	1.97(0.70)	2.01(0.55)	0.845	0.472
**A-o-A**	3.99(0.69)	3.81(0.86)	3.74(0.80)	3.62(0.70)	1.308	0.275
**Wo-F**	80.61(7.89)	76.52(9.17)	81.22(7.92)	79.83(9.14)	1.979	0.120

N-A, Naming Agreement; Im-A, Image agreement; Fam, Familiarity; Vi-C, Visual Complexity; Im-V, Image variability; A-o-A, Age of Acquisition; Wo-F, Word Frequency; SD, Standard deviation.

The reason why we chose three objects was to exclude confounding factors. As many studies have shown that there are significant neural differences between small (<3 or 4) and large numbers (>3 or 4) (Jevons, 1871; Piazza et al., 2003), which may be related to attention (Sophiana and Crosby, 2008) and visuospatial working memory (Luck and Vogel, 1997). If the plural number selection is greater than 3 or 4, the final result may be affected by differences in neural processing of small and large numbers. Therefore, in this study, 3 which can be identified at a glance was selected as the plural number (such as l vs. 3).

#### Procedure

In a sound-attenuated dimly lit chamber, the participants were put on an electrode cap of Ag-AgCl and sat about 120 cm away from a 23-in computer monitor. Eyes were on the same horizontal line as the center of the screen, avoiding excessive eye movements. Stimuli were presented against a dark gray background by the MindXP software developed by our lab, and participants were asked to name the objects rapidly. Meanwhile, the voice by a microphone and EEG were recorded. Before the experiment, participants were pre-tested to ensure that they knew the exact name of the 66 objects. Additional 5 pictures were arranged as a pre-experiment to familiarize participants with the experimental process.

The experiment consisted of two blocks and continued for 6.46 min. One block required approximately 2.7 min to display all the 66 different pictures. One minute was set for a rest between blocks. That is to say, this experiment contains a total of 132 trials, single and plural pictures were 66 trials, respectively. As Figure 1 presented a block began with a fixation cross displayed in the center of the screen for 500 ms. Then pictures for naming were displayed for 1,200 ms, followed by blank screens for a random duration between 1,000 and 1,500 ms to avoid psychological expectations. The 66 pictures were represented in pseudo-random orders: three pictures with similar pronunciation or the same type (singular or plural) did not appear consecutively.

#### Electrophysiological recordings

The EEG recording system was provided by Nanfang Hospital, Southern Medical University, with a 19-channel EEG amplifier (Symptom Instrument^®^). It used an international 10–20 system with linked earlobes as the reference (FP1, FP2, F3, F4, C3, C4, P3, P4, O1, O2, F7, F8, T3, T4, T5, T6, Fz, Cz, and Pz). EEG was continuously recorded at a sampling rate of 1,000 Hz. Recording bandwidth was set at 0.5 to 100 Hz. Electrode impedances were kept below 10 kΩ.

### Data analyses

#### Behavioral analyses

Accuracy and reaction times (RTs) were recorded for each participant by a vocal response using Cool Edit Pro 2.1. The error picture naming included no response (including unnamed and RT over 1,200 ms), word error, and fluency error. And the mean RTs were calculated based on the correct trials. Data were compared between the singular and plural groups using two-tailed paired *t*-tests. Data analysis was performed by SPSS 22.0 software.

#### Event-related potentials analyses

MindWave-sorting software and statistical parametric mapping (SPM) software developed by our lab were used for ERP analyses (application in literature, Zhou et al., 2004, 2019; Cheng et al., 2021). MindWave-sorting software was used for the pre-processing of the EEG data, including automatic correction and ERP extraction. After detection of the ocular, muscular, and any other artifacts at the threshold of ± 70 μV, MindWave-sorting software automatically corrected the EEG signal using principal component analysis (Lins et al., 1993a,b). Then, the epochs were segmented, ranging from −100 ms to 600 ms after stimulus onset, with a baseline correction (using the mean amplitude of 100 ms pre-stimulus interval). Here two ERPs were obtained (singular and plural ERPs) in 19 channels. SPM software was used to obtain the average waveform for each ERP. A pairwise comparison for the two ERPs was performed using two-tailed paired *t*-tests, where correction for multiple testing on the 19 channels was based on the false discovery rate procedure (FDR, Benjamini and Yekutieli, 2001; Lage-Castellanos et al., 2010). And the differences were presented as a topographical map using an interpolation method relevant to a generalized cortical imaging technique (Zhou et al., 1998). A fixed sliding step of 20 ms without overlapping data was set for the topographical map. And 0.05 was set as the significance threshold.

### Results

#### Behavioral data

The overall accuracy was near ceiling (94.98 ± 2.02%) for a total of 132 stimuli. The mean RTs from the onset of pictures to the pronunciation was 612.23 ± 80.02 ms. Specific descriptive statistics of the accuracy and RTs were presented in Table 2, and the paired *t*-test showed no significant differences between the two types of picture naming in both the accuracy and RTs. The error rates (in%) were reported in Supplementary Table 1.

**TABLE 2:** Behavioral performance summary in Experiment 1 (mean ± SD) (*N* = 20).

Behavioral performance	Singular picture naming	Plural picture naming	*t*	*p*
Accuracy (%)	95.05 ± 1.99	94.90 ± 2.10	0.23	0.818
Reaction times (ms)	597.10 ± 79.10	627.36 ± 80.01	−1.20	0.236

#### Waveform and component analysis

**FIGURE 2:**
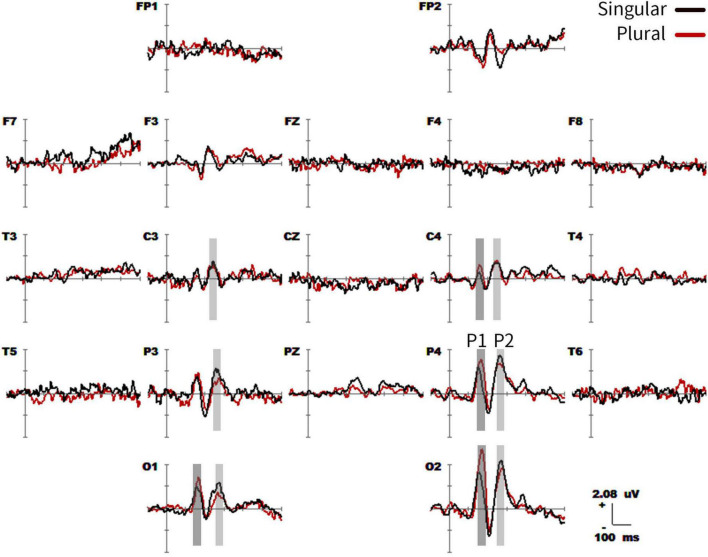
Grand average event-related potential (ERP) waveforms (from –100 to 600 ms) are shown for 19 electrodes across singular (black traces) vs. plural (red traces) picture naming, from the 20 subjects. The baseline ERP measurement is the mean amplitude of a 100-ms pre-stimulus interval.

The grand-average ERPs time-locked to the content word (from −100 to 600 ms) for all the 19 electrodes (FP1, FP2, F3, F4, C3, C4, P3, P4, O1, O2, F7, F8, T3, T4, T5, T6, Fz, Cz, and Pz) were shown in Figure 2. Two phases (P1 effects of 160–180 ms and P2 effects of 220–260 ms in parietal-occipital lobe) showed significant differences in the waveform, but they were consistent after 300 ms. In the first phase (P1 effects), the plural type exhibits greater average amplitudes than the singular type at O1, O2, P4, C4, and T4 electrodes in the range of 160–180 ms. In the second phase (P2 effects), the waveform showed a higher positive average amplitude in the singular type than the plural type at O1, O2, and P3 electrodes within the range of 220–260 ms. All the specific statistics at typical electrodes within a 20-ms time window were shown in Table 3.

**TABLE 3:** Significant waveform effects in 19 channels within a 20-ms time window in Experiment 1 (*N* = 20).

Effect	P1 (O2)		P1 (P4)		P1 (C4)		P1 (T4)		P1 (O2)	
	Stat.	*p*/WO	Stat.	*p*/WO	Stat.	*p*/WO	Stat.	*p*/WO	Stat.	*p*/WO
*t/p*	−3.52	0.002	−2.79	0.012	−2.37	0.034	−2.66	0.017	−2.16	0.044
Cohen’s d/WO	−1.62	160	−1.28	160	−1.09	160	−1.22	160	−0.99	180
Effect	P2 (O1)		P2 (O1)		P2 (O2)		P2 (P3)		P2 (O1)	
	Stat.	*p*/WO	Stat.	*p*/WO	Stat.	*p*/WO	Stat.	*p*/WO	Stat.	*p*/WO
*t/p*	2.75	0.013	2.15	0.049	2.53	0.020	2.44	0.026	2.14	0.046
Cohen’s d/WO	1.26	220	0.99	240	1.16	240	1.12	240	0.98	260

Significant waveform effects, electrodes and time windows (20 ms interval as a time window) with significant amplitude differences of singular vs. plural ERPs within 0–600 ms, and their corresponding maximum statistics (t, p, and Cohen’s d); P1 (O2), P1 effect (electrode); P2 (O2), P2 effect (electrode); Stat., statistics; p, FDR-corrected p-value of paired T-test; WO, window set (at 20 ms intervals). WO, window onset.

#### Spatiotemporal pattern: SPM (t)

**FIGURE 3:**
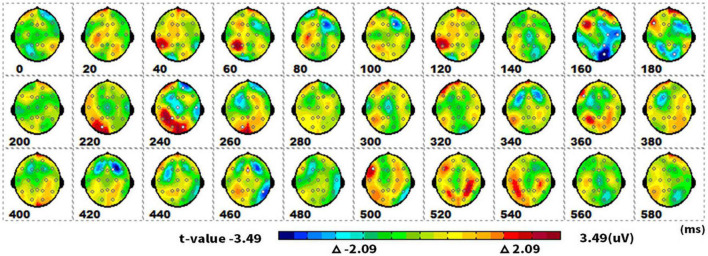
The spatiotemporal patterns of SPM (t) (0 to 600 ms) are derived from the two-tailed paired *t*-tests (singular vs. plural). Each map was interpolated from the average *t*-values within the fixed 20-ms time window, and the red/bright blue bin of the color scale corresponded to the 0.05 significance threshold: *t*_(1,19)_ = ± 2.09. The white dots represented the electrode sites with significant effects.

Figure 3 showed topographical maps of SPM (t) (0–600 ms) derived from two-tailed paired *t*-tests. The red/bright blue bin of the color scale corresponded to the 0.05 significance threshold: *t*_(1_,_19)_ = ± 2.09; the white dots on the maps represented the electrode sites with significant effects. The two types of pictures initially showed differences in neural processing at the parietal-occipital lobe of 160–180 ms. As the neural processing progressed, they differed at 220–260 ms in the parietal-occipital lobe again.

### Discussion

Stimulus items were matched for familiarity and naming accuracy of image materials of the two sets of pictures. As anticipated, naming accuracy was found to be very high for singular and plural pictures, with no significant difference. Besides, no significant difference was found in the RTs between the two types of pictures. However, based on ERP results, the two types of pictures showed differences in amplitude in two effects (parietal-occipital P1 and P2) within the first 300 ms of picture naming. Therefore, using behavioral data to determine the difference between singular and plural picture naming may be insufficient.

To the best of our knowledge, this is the first study to compare neural processes of Chinese singular and plural picture naming. Given that word production of Chinese singular and plural nouns is consistent, we suggest that ERP differences between the two types of pictures (P1 and P2 effects) may represent the differences in quantitative processing (singular vs. plural). First, study proved that the EEG signal was not contaminated by speech artifacts up to 100 ms before articulation (Fargier et al., 2017). In this study, the ERP differences were in 0–300 ms, while pronunciation was around 650 ms, so P1 and P2 effects would not be interfered by pronunciation. Second, the P1 effect was not related to visual perception. Visual perception is an exogenous component and generally differs within 100 ms, while the P1 effect was after 100 ms. Third, ERP differences cannot be explained by the differences in singular and plural word production. In word production, 160–180, and 220–260 ms periods represent lexical retrieval, lemma selection, and phonological coding, respectively (Hassan et al., 2015). Accordingly, the activated brain areas gradually transition from the back to front, from the occipital lobe to the inferior temporal sulcus and the frontal area. However, in the present study, both effects of ERPs differed in the parieto-occipital lobe. Fourth, the ERP differences were not inflection. Inflection occurred in the left inferior frontal gyrus at 280–400 ms, with a peak at 320 ms (Sahin et al., 2009). In this study, the ERPs of the two types of pictures showed no difference after 260 ms. It also suggests that Chinese picture naming lacks inflection processing. Last but not least, the ERP differences in this study were basically consistent with the previous literature on the time course, amplitude features, and activated brain regions of quantitative processing ERPs (Libertus et al., 2007; Pinhas et al., 2015). The first ERP difference (P1 effect of 160–180 ms in parietal-occipital lobe) had a larger amplitude as a larger quantity, which conforms to the characteristics of the summation coding. The second ERP difference (P2 effect) was in the parieto-occipital lobe at 220–260 ms and corresponded to the spatial coding. Singular images got larger amplitudes. The reason may be that in the daily life the picture naming is always based on a singular picture. And the number one was closer to the participants’ psychological preference number (Libertus et al., 2007; Nieder and Merten, 2007; Pinhas et al., 2015). However, the summation coding was a positive effect in our study and a negative effect in the previous literature (Libertus et al., 2007; Pinhas et al., 2015). We considered that the difference in polarity of this effect is due to the different experimental tasks (picture naming vs. counting task). Picture naming affected the ERP waveform of summation coding.

The neural processes of Chinese singular and plural picture naming are different. It supports our hypothesis that in the act of singular and plural picture naming, there are two simultaneous neuropsychological processes: word production and quantitative processing. Meanwhile, both neural processes showed electrical activity in the parieto-occipital lobe at 140–220 ms. Whether this is a mere coincidence or there is a certain connection between these two neural processes warrants further investigation. Therefore, Experiment 2 was designed to explore this issue.

## Experiment 2

According to the “Lemma model,” picture naming goes through two psychological processes from visual feature extraction to lexical concept formation/semantic formation: concept gathering (color, shape, movement, motion features, hearing, smell, taste, etc.) and viewpoint selection (relative relation within objects). The relative relationship can be orientational, quantitative, etc. That is, semantic formation contains quantitative information. Conversely, quantity differences of picture naming can also be expressed in semantics and vocabulary (for example, basketball vs. basketballs). Therefore, it is reasonable to hypothesize that semantic and quantitative information in singular and plural picture naming may have a certain connection. Based on this, they all showed electrical activity in the parieto-occipital lobe at 140–220 ms, corresponding to the semantic formation and summation encoding, respectively. Hence, Experiment 2 was designed by taking Chinese as the target language and controlling semantics (S) and quantity (Q) as two factors, with two levels: inanimate (S−) vs. animate (S +) and singular (Q−) vs. plural (Q +), to explore the correlation between semantic and quantity factors using a 2 × 2 variance analysis.

### Study design

#### Participants

According to the standard of Experiment 1, another 25 postgraduate students (12 male; aged 18–29 years old, and mean age = 24.1 years old, SD: 3.09 years) completed the experiment.

#### Stimuli

Following the method in Experiment 1, 132 pictures with different objects were prepared. Four conditions (S−Q−, S + Q−, S−Q +, S + Q +) each had 33 pictures. And the inanimate singular and plural pictures (S−Q−, S−Q +) were exactly the same as in Experiment 1.

#### Procedure

**FIGURE 4:**
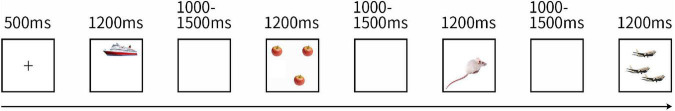
Task Design of Experiment 2. The picture shows the design of the experimental task. All trials followed the depicted sequence. A block began with a fixation cross a picture. Then a singular – inanimate/plural – animate/singular – inanimate/plural – animate picture was displayed and participants were asked to name the objects rapidly after the stimuli were presented, followed by a blank screen (three pictures with similar pronunciation or the same quantity (singular vs. plural)/semantics (animate vs. inanimate) did not appear consecutively).

The experiment was performed as previously described in Experiment 1. The task was still to name the pictures as quickly as possible. The experiment consisted of 264 trials, with 66 trials for each of the four conditions, and divided into 4 blocks (2.7 min each). The stimulus was shown in Figure 4, using the same method as Experiment 1, but with the added restriction that no more than three inanimate or animate pictures could be seen sequentially.

#### Electrophysiological recordings

Electrophysiological recordings were the same as in Experiment 1.

### Data analyses

#### Behavioral analyses

The accuracy and RTs of picture naming were analyzed by a repeated-measures ANOVA.

#### Event-related potentials analyses

The software and methods of ERP processing and analysis were similar to those applied in Experiment 1. Since it was likely established in Experiment 1 that quantitative processing occurred before 300 ms, semantic processing of naming also ended before this period. The waveforms and topographic maps of ERP in Experiment 2 were intercepted from −100 to 400 ms. Eventually, Experiment 2 had 4 ERPs (S−Q−, S + Q−, S−Q +, S + Q +) in 19 channels, segmented within 0 to 400 ms. The within-subject factors were semantic (animate vs. inanimate) and quantity factors (singular vs. plural). Then a two-way repeated-measures ANOVA was performed on the four variables, with multiple testing on the 19 channels corrected using the FDR procedure (Benjamini and Yekutieli, 2001; Lage-Castellanos et al., 2010). Similarly, the differences were represented by a topographical map with a fixed sliding window of 20 ms, and the white dots on maps indicated significant effects.

### Results

#### Behavioral data

The overall naming accuracy of all 264 stimuli was very high, approximately 94.87 ± 1.61%. The mean RTs were 649.17 ± 113.85 ms. Table 4 described the specific values of naming accuracy and RTs of the four conditions. And the results of the repeated-measures ANOVA, which indicated that there was no significant difference in behavioral performance among the four conditions, were presented in Table 5. The error rates were reported in Supplementary Table 2.

**TABLE 4:** Behavioral performance summary in Experiment 2 (mean ± SD) (*N* = 24).

	Reaction times (ms)		Accuracy (%)	
	Singular	Plural	Singular	Plural
Animate pictures	644.56 ± 112.80	667.33 ± 105.86	94.71 ± 1.27	95.46 ± 1.77
Inanimate pictures	640.34 ± 127.18	644.46 ± 116.40	94.67 ± 1.55	94.63 ± 1.79

**TABLE 5:** Two-factor ANOVA of repeated measures of behavioral data (*N* = 24).

	Reaction times		Accuracy	
	*F*	*P*	*F*	*p*
Semantic	0.328	0.568	1.777	0.186
Quantitative	0.323	0.571	1.164	0.283
Interaction	0.155	0.694	1.454	0.231

#### Waveform and component analysis

**FIGURE 5:**
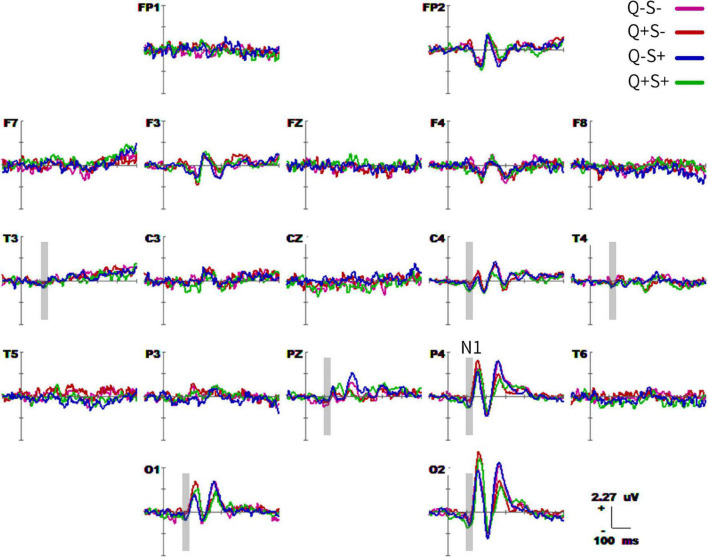
Grand average event-related potential (ERP) waveforms (from –100 to 600 ms) are shown for 19 electrodes across four trial types. The purple, red, blue, and green traces correspond to group average ERP of the singular – inanimate (Q–S–), plural- – inanimate (Q + S–), singular – animate (Q–S +), and plural-animate (Q + S +) conditions, respectively. The baseline ERP measurement is the mean amplitude of a 100-ms pre-stimulus interval.

The grand-average waveforms of the four ERPs (−100 to 600 ms) are shown in Figure 5. There were differences in the waveforms of singular and plural pictures (whether they are animate or inanimate) in the parieto-occipital lobe (O1, O2, P3, P4, and PZ) at 160–180 ms (P1 effect), and parieto-occipital lobe (O1, O2, P4, and PZ) at 220–260 ms (P2 effect). The difference of waveforms between animate and inanimate pictures (regardless of singular and plural factors) was in the parieto-occipital temporal lobe (O1, O2, P3, P4, T5, T6, T3, T4, and F7) at 100–140 ms (N1 effect). And the animate pictures got larger N1 than the inanimate pictures. Table 6 detailed the average statistics of the waveforms at typical electrodes.

**TABLE 6:** Significant waveform effects in 19 channels within a 20-ms time window in Experiment 2 (*N* = 24).

Effect	*F*(1,23)/*p*	P1 (O1)		P1 (O2)		P1 (Pz)		P1 (P3)		P1 (P4)	
	ES/WO	Stat.	*p*/WO	Stat.	*p*/WO	Stat.	*p*/WO	Stat.	*p*/WO	Stat.	*p*/WO
Quantitative	F/*p*	9.67	0.006	11.34	0.003	5.96	0.036	11.32	0.003	10.16	0.004
	η2p/WO	0.30	160	0.33	160	0.21	160	0.33	160	0.31	160
	*F*(1,23)/*p*	P1 (O1)		P1 (O2)		P1 (P3)		P2 (O1)		P2 (O2)	
	ES/WO	Stat.	*p*/WO	Stat.	*p*/WO	Stat.	*p*/WO	Stat.	*p*/WO	Stat.	*p*/WO
Quantitative	F/*p*	6.64	0.02	4.54	0.044	10.85	0.003	13.36	0.001	24.92	0.000
	η2p/WO	0.22	180	0.16	180	0.32	180	0.37	220	0.52	220
	*F*(1,23)/*p*	P2 (Pz)		P2 (P4)		P2 (O1)		P2 (O2)		P2 (Pz)	
	ES/WO	Stat.	*p*/WO	Stat.	*p*/WO	Stat.	*p*/WO	Stat.	*p*/WO	Stat.	*p*/WO
Quantitative	F/*p*	20.94	0.010	11.8	0.003	12.3	0.002	23.3	0.000	21.07	0.000
	η2p/WO	0.48	220	0.34	220	0.35	240	0.50	240	0.48	240
	*F*(1,23)/*p*	P2 (P4)		P2 (O2)		P2 (Pz)		P2 (P4)		P2 (O2)	
	ES/WO	Stat.	*p*/WO	Stat.	*p*/WO	Stat.	*p*/WO	Stat.	*p*/WO	Stat.	*p*/WO
Quantitative	F/*p*	11.5	0.004	10.40	0.010	7.44	0.013	4.91	0.044	4.79	0.041
	η2p/WO	0.33	240	0.31	260	0.24	260	0.18	260	0.17	280
	*F*(1,23)/*p*	N1 (O1)		N1 (O2)		N1 (P4)		N1 (C4)		N1 (F7)	
	ES/WO	Stat.	*p*/WO	Stat.	*p*/WO	Stat.	*p*/WO	Stat.	*p*/WO	Stat.	*p*/WO
Semantic	F/*p*	10.59	0.003	5.90	0.034	11.28	0.003	8.79	0.008	6.08	0.03
	η2p/WO	0.32	100	0.20	100	0.33	100	0.28	100	0.21	100
	*F*(1,23)/*p*	N1 (F4)		N1 (O2)		N1 (T5)		N1 (P4)		N1 (T5)	
	ES/WO	Stat.	*p*/WO	Stat.	*p*/WO	Stat.	*p*/WO	Stat.	*p*/WO	Stat.	*p*/WO
Semantic	F/*p*	8.31	0.01	4.99	0.048	12.5	0.002	10.3	0.004	4.76	0.0475
	η2p/WO	0.27	100	0.18	120	0.35	120	0.31	120	0.17	140
	*F*(1,23)/*p*	N1 (P3)		N1 (T6)							
	ES/WO	Stat.	*p*/WO	Stat.	*p*/WO						
Semantic	F/*p*	6.02	0.025	10.13	0.004						
	η2p/WO	0.21	140	0.31	140						
	*F*(1,23)/*p*	Cz		Cz		Cz		Cz		Cz	
	ES/WO	Stat.	*p*/WO	Stat.	*p*/WO	Stat.	*p*/WO	Stat.	*p*/WO	Stat.	*p*/WO
Interaction	F/*p*	7.35	0.012	5.78	0.025	7.31	0.013	9.47	0.005	7.62	0.011
	η2p/WO	0.24	180	0.20	200	0.24	220	0.29	240	0.25	280

Significant waveform effects, electrodes and time windows (20 ms interval as a time window) with significant amplitude differences of singular vs. plural/inanimate vs. animate/interaction ERPs within 0–600 ms, and their corresponding maximum statistics (F, *p*, and η2p); Stat., statistics; p, FDR-corrected F-value of ANOVA; WO, window set (at 20 ms intervals).

#### Spatiotemporal pattern: SPM (f)

**FIGURE 6:**
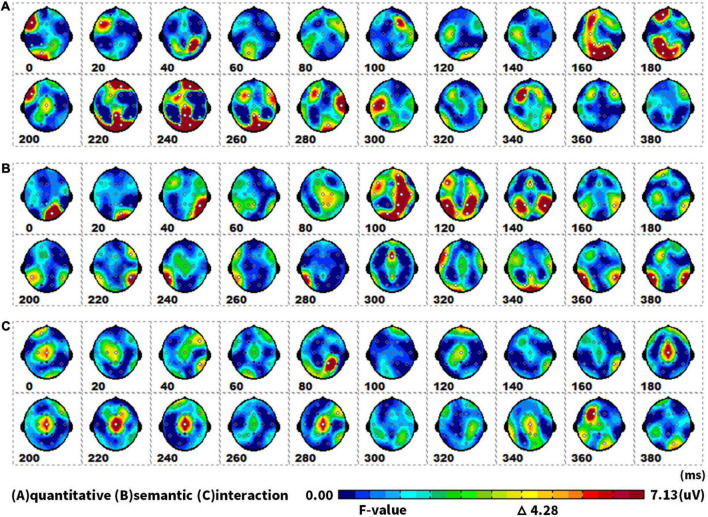
The spatiotemporal patterns of SPM (f) (0 to 400 ms) are derived from the two-way (semantic: animate vs. inanimate, and quantitative: singular vs. plural) repeated measures ANOVA: **(A)** the quantitative effect, **(B)** the semantic effect, and **(C)** the interaction effect. Each map was interpolated from the average *F*-values within the fixed 20-ms time window, and the bright yellow bin of the color scale corresponded to the 0.05 significance threshold: *F*_(1,23)_ = 4.28. The white dots represented the electrode sites with significant effects.

Figure 6 showed topographical maps of SPM (f) (0 to 400 ms), which were derived from the two-way repeated-measures ANOVA of waveforms. Figure 6A indicated the main effect of quantity. The quantity factor (singular vs. plural) led to significant differences in two ERPs: the first in the parieto-occipital lobe at 160–180 ms, and the second in the parieto-occipital area at 220–260 ms. Figure 6B showed that semantic processing (animate vs. inanimate) mainly induced an ERP difference in the parieto-occipital temporal area at 100–140 ms. Figure 6C revealed that semantic and quantity factors in picture naming have an interactive effect at 180–280 ms in the central area.

**FIGURE 7:**
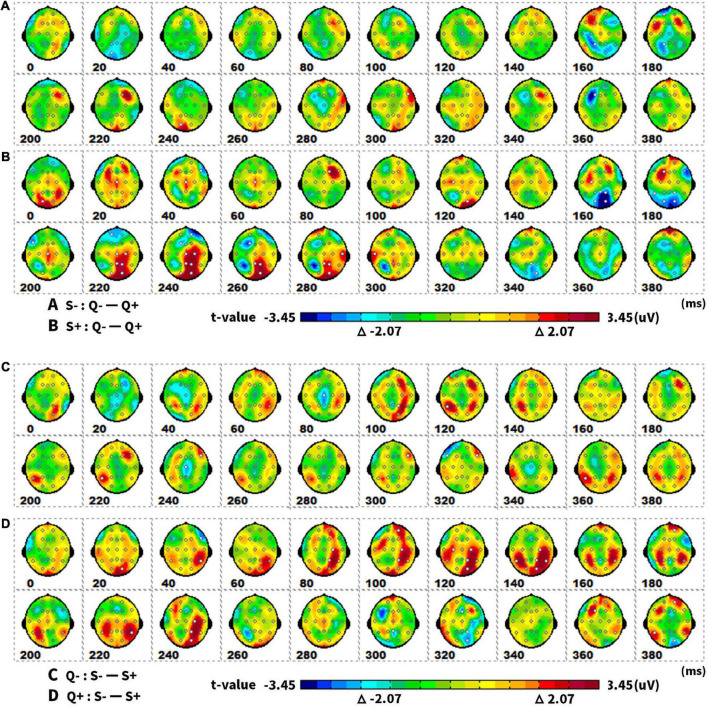
The spatiotemporal patterns of SPM (t) (0 to 400 ms) are derived from the pairwise comparisons between the conditions (Q–, Q +, S–, S +): **(A)** (S–. Q– – Q +), **(B)** (S +. Q– – Q +), **(C)** (Q–. S– – S +), **(D)** (Q +. S– – S +). Each map was interpolated from the average *t*-values within the fixed 20-ms time window, and the red/bright blue bin of the color scale corresponded to the 0.05 significance threshold: *t*(1,23) = ± 2.07. The white dots represented the electrode sites with significant effects. Q–, singular; Q +, plural; S–, inanimate; S +, animate.

The results of the *post hoc* tests were presented as topographic maps in Figure 7. The ERP differences of singular and plural picture naming – the amplitude differences in the parieto-occipital area at 160–180 ms and 220–280 ms were larger in the animate items (S + : Q− – Q +) than that in the inanimate items (S−: Q− – Q +). The plural items (Q + : S− – S +) got greater ERP differences in animate and inanimate picture naming than the singular items (Q−: S− – S +). The ERPs differed not only in the parieto-occipital temporal area at 100–140 ms but also in the parieto-occipital area at 160–180 ms and 220–280 ms.

### Discussion

The naming accuracies and RTs of singular vs. plural pictures in Experiment 2 were similar to those in Experiment 1. And semantic and quantitative differences did not have significant effects on behavioral results. Nonetheless, this study revealed that semantic and quantity factors of Chinese singular and plural picture naming interact in the central area at 180–280 ms using ERP data. It is supported by some research in recent years that semantics interact with quantitative processing. It was found that the peripheral region of the lateral and medial parietal cortex in semantic networks is selective for numbers (Huth et al., 2016). Meanwhile, an fMRI study found that quantifiers were identical to the processing of animal names (Wei et al., 2014). Another fMRI study suggested that calculation and language processes shared a common neural substrate since both of them activated the temporal lobe (Zago et al., 2008). However, the two tasks were separate procedures, and the idea of common neural substrates is not convincing. In our study, the interaction of semantic and quantity factors is unified in time and space owing to the one task and 2 × 2 variance analysis adopted.

Event-related potentials results showed that the main effect of the quantitative factor in Experiment 2 is consistent with the differences of singular and plural picture naming in Experiment 1, confirming that there was spontaneous quantitative processing at picture naming. The main effect of the semantic factor is also consistent with previous literature, manifested in the parieto-occipital temporal area at 100–140 ms. Semantic and quantitative factors affect each other in the process of picture naming, with a significant interaction at 180–280 ms. According to the time nodes of semantic, quantitative, and interactive processing, we considered that the summation coding and semantic processing interact with each other. And they affect the lexical retrieval, lemma selection, and integrating access to phonological forms of picture naming (Hassan et al., 2015). This is consistent with the differences in English singular and plural nouns (words and pronunciation). *Post hoc* tests showed that the difference in quantitative processing of animate pictures was larger than that of inanimate pictures. Plural pictures have greater differences in semantic and quantitative processing than singular pictures. This also indicates that semantics interact with quantitative processing. Although there was no statistical difference in behavior results among the four conditions, the RTs of the plural animate pictures were the largest. And this may be related to increased cognitive load.

## General discussion

Because the vocabularies/word production processes of Chinese singular and plural picture naming were the same, it is often overlooked that the neural processing process of these two types of picture naming may be inconsistent. This paper investigated the neuropsychological processes of Chinese singular and plural picture naming. “Experiment 1” revealed that the neural electrical activities of these two types of picture naming were different. The ERP differences between the two conditions could not be explained by word production, but were close to the ERP differences in different quantities. It indicates that the Chinese singular and plural picture naming is not only a program of word production but also may involve quantitative processing. “Experiment 2” further explored that these two processes are not isolated, and there is an interaction between them.

Although we explored the neuropsychological processes associated with single and plural picture naming and highlighted the theories of picture naming for the first time, this study has several limitations. First, in this study, we only choose 1 and 3 which were both small numbers for the comparison of singular and plural numbers. In the future, different plural numbers should be tested to provide additional evidence for the neural mechanism of picture naming (such as 5, 6, etc.). Second, in the present study, singular and plural pictures had the same visual complexity. If objects of single and plural images were set to the same size, more information about quantitative processing could be obtained. Third, the target language used for picture naming in the present study was Chinese, and given the universality of neural processes involved in picture naming with regard to language, the present findings should be validated in different languages.

## Conclusion

This is the first study to investigate neuropsychological processes associated with singular and plural picture naming in the Chinese language. Results showed that singular and plural picture naming may involves two simultaneous neural processes: word production and quantitative processing. Moreover, these two processes share a common neural substrate – they interact at 180–280 ms in the central area.

## Data availability statement

The raw data supporting the conclusions of this article will be made available by the authors, without undue reservation.

## Ethics statement

The studies involving human participants were reviewed and approved by the Medical Ethics Committee of the First Affiliated Hospital of Jinan University. The patients/participants provided their written informed consent to participate in this study.

## Author contributions

L-YC and W-WC contributed to the conception and design of the study and performed the writing of this manuscript. L-YC, S-RS, C-MS, and RL acquired the recording data. SZ and Z-MC revised the main manuscript text. S-YB and WL modified the article.
