# Supplementary material for: Spontaneous quantitative processing in Chinese singular and plural picture naming: An event-related potentials analysis
Source: Front Neurosci. 2022 Oct 11;16:898526. doi: 10.3389/fnins.2022.898526 (PMC9594987; doi:10.3389/fnins.2022.898526)
Supplement: Supplementary file 1 [file Table_1.DOC]

**Appendix 1.** Stimuli used in the experiment.

1. S-, singular－non-living pictures;

Q+S-, plural－non-living pictures;

Q-S+, singular－living pictures;

Q+S+, plural－living pictures;

Q-S- and Q+S- were both used in Experiment 1 and 2.

| **Q-S-** | | **Q-S+** | | **Q+S-** | | **Q+S+** | |
| --- | --- | --- | --- | --- | --- | --- | --- |
| Chinese name | English name | Chinese name | English name | Chinese name | English name | Chinese name | English name |
| 算盘 | Abacus | 蜜蜂 | Bee | 地球仪 | Globe | 鳄鱼 | Crocodile |
| 斧头 | Axe | 珊瑚 | Coral | 电熨斗 | Clothes iron | 青蛙 | Frog |
| 注射器 | Syringe | 海龟 | Turtle | 拖拉机 | Tractor | 火龙果 | Pitaya |
| 毛笔 | Ink brush | 羊 | Sheep | 鼓 | Drum | 猪 | Pig |
| 篮球 | Basketball | 鹿 | Deer | 钢琴 | Piano | 藕 | Lotus root |
| 飞机 | Airplane | 梨 | Pear | 轮船 | Ship | 虾 | Shrimp |
| 小提琴 | Violin | 辣椒 | Pepper | 手风琴 | Accordion | 哈密瓜 | Hami melon |
| 坦克 | Tank | 熊 | Bear | 笛子 | Dizi | 马 | Horse |
| 平底锅 | Flying pan | 袋鼠 | Kangaroo | 吸尘器 | Vacuum cleaner | 仙人掌 | Cactus |
| 菜刀 | Kitchen Knife | 鲨鱼 | Shark | 镰刀 | Sickle | 乌龟 | Turtle |
| 勺子 | Spoon | 猫 | cat | 弓箭 | Bow and arrow | 猴子 | Monkey |
| 剪刀 | Scissors | 乌贼 | Cuttlefish | 铲子 | Shovel | 老鼠 | Rat |
| 二胡 | Erhu | 螃蟹 | Crab | 手枪 | Handgun | 鸭子 | Duck |
| 跳绳 | Skipping rope | 狗 | dog | 叉子 | Fork | 鲸鱼 | Whale |
| 针管 | Syringe | 樱桃 | Cherry | 足球 | Football | 金鱼 | Goldfish |
| 牙膏 | Toothpaste | 蜘蛛 | Spider | 别针 | Safety pin | 鹦鹉 | Parrot |
| 圆规 | Compass | 橙子 | Orange | 滑板 | Skateboard | 蝴蝶 | Butterfly |
| 电视机 | TV | 柠檬 | Lemon | 放大镜 | Magnifying glass | 蚂蚁 | Ant |
| 电钻 | Electric drill | 葡萄 | Grape | 衣柜 | Wardrobe | 香瓜 | Mushmelon |
| 卷尺 | Tape measure | 石榴 | Pomegranate | 篮子 | Basket | 芒果 | Mango |
| 收音机 | Radio | 毛毛虫 | Caterpillar | 手电筒 | Flashlight | 西红柿 | Tomato |
| 古筝 | Guzheng | 木瓜 | Papaya | 香烟 | Cigarette | 柿子 | Persimmon |
| 羽毛球 | Badminton | 青椒 | Green pepper | 电热毯 | Electric blanket | 竹笋 | Bamboo shoot |
| 火车 | Train | 西瓜 | Watermelon | 墨水 | Ink | 桃子 | Peach |
| 灭火器 | Fire extinguisher | 黄瓜 | Cucumber | 水龙头 | Tap | 苹果 | Apple |
| U盘 | USB | 土豆 | Potato | 卡车 | Truck | 菊花 | Chrysanthemum |
| 背心 | Sleeveless shirt | 莴笋 | Celtuce | 镊子 | Tweezers | 南瓜 | Pumpkin |
| 唢呐 | Suona | 荷花 | Lotus flower | 光盘 | Optical disc | 蘑菇 | Mushroom |
| 钉子 | Nail | 萝卜 | Radish | 铅笔 | Pencil | 菜花 | Cauliflower |
| 剑 | sword | 蛇 | snake | 锣 | Gong | 牛 | cow |
| 色子 | Dice | 榴莲 | Durio zibethinus | 拨浪鼓 | Rattle | 核桃 | Walnut |
| 方向盘 | Steering wheel | 牡丹花 | Moutan peony | 三角尺 | Trianglar ruler | 玫瑰花 | Rose |
| 口琴 | Harmonica | 椰子 | Coconut | 胶棒 | Glue stick | 豌豆 | Pea |
