# Supplementary material for: Spontaneous quantitative processing in Chinese singular and plural picture naming: An event-related potentials analysis
Source: Front Neurosci. 2022 Oct 11;16:898526. doi: 10.3389/fnins.2022.898526 (PMC9594987; doi:10.3389/fnins.2022.898526)
Supplement: Supplementary file 2 [file Table_2.DOC]

Table S1. Error rates for each error type of Experiment 1 (mean ± SD) (N = 20).

| Type of erro  Task | Singular picture naming | Plural picture naming |
| --- | --- | --- |
| no response (%) | 1.80 ± 1.24 | 1.90 ± 1.17 |
| word error (%) | 1.20 ± 0.70 | 1.10 ± 0.91 |
| fluency error (%) | 1.95 ± 1.39 | 2.15 ± 1.46 |

Table S2. Error rates for each error type of Experiment 2 (mean ± SD) (N = 24).

| Type of error  Task | Living pictures | | Non-living pictures | |
| --- | --- | --- | --- | --- |
|  | Singular | Plural | Singular | Plural |
| no response (%) | 2.00 ± 0.59 | 1.75 ± 0.74 | 2.00 ±0.72 | 2.25 ±0.68 |
| word error (%) | 1.63 ± 0.77 | 1.25 ± 0.99 | 1.42 ± 1.02 | 1.33 ± 1.13 |
| fluency error (%) | 1.67 ± 0.70 | 1.54 ± 1.02 | 1.92 ± 1.02 | 1.79 ± 1.22 |
